# Unique and shared influences of anxiety and ADHD on the behavioral profile of autism in early childhood

**DOI:** 10.3389/frcha.2025.1585507

**Published:** 2025-06-05

**Authors:** Diana K. Waters, Grace T. Baranek, Elizabeth Glenn, Hannah Riehl, Lauren DeMoss, Geraldine Dawson, Kimberly L. H. Carpenter

**Affiliations:** ^1^ Duke Center for Autism & Brain Development, Department of Psychiatry and Behavioral Sciences, Duke University School of Medicine, Durham, NC, United States; ^2^ Department of Allied Health Sciences, University of North Carolina at Chapel Hill, Chapel Hill, NC, United States

**Keywords:** autism, executive function, anxiety, ADHD, pediatric

## Abstract

**Introduction:**

Autism is characterized by a wide range of core and associated behavioral features that can be influenced by co-occurring conditions such as attention-deficit hyperactivity disorder (ADHD) and anxiety disorders. Executive function difficulties are proposed as a common feature of autism and ADHD and are also evident in persons with anxiety disorders. However, little is known about how anxiety disorders or ADHD differentially impact executive functioning or how these difficulties may influence the presentation of core and associated autistic features in young children. In the current study, we explored the unique executive function difficulties associated with co-occurring anxiety and/or ADHD and elucidated how they differentially impact the clinical presentation of autism in young children.

**Methods:**

We assessed 69 autistic children, aged 3 to 5 years. Anxiety and ADHD were assessed through parent interview using the Preschool Age Psychiatric Assessment (PAPA). Executive functions were assessed using the Behavior Rating Inventory of Executive Function – Preschool Version (BRIEF-P). Core autistic features were measured with the Autism Diagnostic Observation Schedule—Second Edition (ADOS-2) and additional features were measured with the Restricted and Repetitive Behaviors Scale, Revised (RBS-R) and the Sensory Experiences Questionnaire (SEQ). Using an additive main effect general linear model, we examined the unique contributions of an anxiety disorder and/or ADHD on core and associated autistic features as well as executive function. Mediation analyses explored the contribution of the executive function profiles to specific features of autism.

**Results:**

Results showed that greater difficulty with attentional shifting was uniquely associated with anxiety, whereas greater difficulty inhibiting behavioral responses was uniquely associated with ADHD. Attentional shifting mediated the relationship between anxiety and ritualistic behaviors, sameness behaviors, sensory hyper-responsivity, and overall autistic features. Conversely, inhibitory control mediated the relationship between ADHD and both irritability and self-injurious behaviors.

**Discussion:**

These findings implicate components of executive functioning as important cognitive processes associated with co-occurring psychiatric conditions in autism. Future research should investigate the impact of early intervention for executive function difficulties on psychiatric and neurodevelopmental outcomes in autistic children.

## 1 Introduction

Autism is a broad neurodevelopmental condition with a wide range of presentations, strengths, and support needs. Autistic individuals can experience several associated features such as differences in sensory perception, difficulties in emotional regulation, impaired sleep, gastrointestinal disturbances, and executive functioning differences. The degree and nature of both core and associated features of autism varies widely and may be influenced by the presence of co-occurring conditions (1, 2). Autistic individuals are more likely to have a mental health condition than non-autistic individuals, estimates suggest approximately 70% of autistic individuals have a co-occurring mental health condition (3–5). Exploring the influence of co-occurring conditions, particularly those presenting in early life, could improve our understanding of the differences among autistic individuals and support more targeted interventions.

The two most common co-occurring psychiatric conditions in autistic individuals are attention-deficit hyperactivity disorder (ADHD) and anxiety disorders. ADHD is present in around 50%–70% of autistic individuals (6). Like autism, ADHD is a neurodevelopmental disorder, however age at diagnosis varies and is impacted by co-occurrence of the two (7, 8). Rates of anxiety diagnoses in autistic children increase with age. Around 20% of autistic preschoolers have an anxiety disorder compared to 9.5% of preschoolers without autism. By school age, 26% of autistic children have an anxiety disorder and by adolescence, this increases to 41% (9). Autistic children with co-occurring ADHD have higher rates of anxiety symptoms than children with only an autism diagnosis (10–14). These findings suggest there are likely influences between these conditions.

Autism, anxiety and ADHD are known to impact executive function, which are the cognitive processes involved in planning and goal execution. Executive function difficulties are proposed as a common feature between autism and ADHD and are also implicated in anxiety. In ADHD, difficulties with inhibitory control are central to the disorder and correlate with autistic traits (15–23). In both autism and anxiety, difficulties with shifting and cognitive flexibility are common (24, 25); however it is still unknown whether the presence of co-occurring anxiety has differential impacts on executive function difficulties in young autistic children. With respect to ADHD, previous work suggests executive difficulties do not differentiate between autism and ADHD, but are associated with increased anxiety and depression in autistic individuals with co-occurring ADHD (26). Despite the high rates of co-occurring ADHD and anxiety in autistic children, relatively few studies have explored how executive functions differ in autistic children with both co-occurring conditions (1 27–28).

In the current study, our aim was to identify influences of co-occurring ADHD and anxiety on executive functioning and core and associated features of autism in young children (ages 3–5 years). Specifically, we hypothesized that ADHD and anxiety will each be associated with unique patterns of executive function difficulties and that those difficulties will mediate the expression of different features of autism.

## 2 Methods

### 2.1 Participants

Participants were 69 children diagnosed with autism spectrum disorder, aged 3 years to 5 years and 11 months, who were part of a larger study exploring sensory processing and anxiety in autistic children. Testing was conducted at the Duke Center for Autism and Brain Development in Durham, NC, between May 2015 and September 2017. Children were eligible for the study if they had been diagnosed with or were suspected of meeting DSM-5 criteria for Autism Spectrum Disorder. Diagnoses were established or confirmed using the Autism Diagnostic Observation Schedule—Second Edition [ADOS-2 (29)], and the Autism Diagnostic Interview—Revised [ADI-R (64)]. Participant demographic characteristics are shown in Table 1.

**Table 1:** Participant demographic and clinical characteristics (*N* = 69).

Characteristic	Value
Age in Years [Mean (SD)]	4.1 (0.9)
Gender [*N* (%)]	
Girls	15 (22%)
Boys	54 (78%)
Ethnicity/Race [*N* (%)]	
Black	5 (7%)
White, Non-Hispanic	30 (43%)
Hispanic	10 (15%)
More than one race	18 (26%)
Other	6 (9%)
Co-occurring Diagnoses [*N* (%)]	
Anxiety Disorders (any)	49 (71%)
Anxiety only	18 (26%)
Anxiety+ADHD	31 (45%)
ADHD (any)	42 (61%)
ADHD only	11 (16%)
Anxiety+ADHD	31 (45%)
Developmental Quotient [Mean (SD)]	77 (33.2)

ADHD, attention-deficit hyperactivity disorder.

Exclusion criteria included known presence of a genetic disorder associated with autism (e.g., Fragile X Syndrome), uncorrected vision or hearing impairments, significant motor impairment (e.g., cerebral palsy), chronic or acute medical illness, having a seizure in the last year, a diagnosis of a seizure disorder, and/or taking medicine to control seizures.

Participants were recruited to the Duke University Center for Autism and Brain Development through various community outreach methods including flyers, brochures, emails, and social media posts, as well as through the center's volunteer research registry. Parents/legal guardians were compensated for their time, and children received a toy at each visit to the center for their participation. All parents/legal guardians consented to participate in the study, and all study procedures were approved by the Duke University institutional review board for research with human subjects.

### 2.2 Measures

#### 2.2.1 Core and associated features

Core autism features, including social and communication difficulties as well as repetitive and restricted behaviors, were evaluated with the ADOS-2. Restricted and repetitive behaviors were further evaluated via the Restricted and Repetitive Behaviors Scale, Revised [RBS-R (30, 31)]. The RBS-R is parent report measure with a 43-item scale that measures specific behaviors and rates the level of severity. Behavioral profiles can be identified by the subscores of stereotyped, self-injurious, compulsive, ritualistic, sameness, and restricted behaviors.

Sensory challenges were measured via the Sensory Experiences Questionnaire version 3.0 [SEQ (32)] a caregiver report measure of the child's response to sensory stimuli in the context of daily activities and routines. The SEQ has been validated for autistic children ages 2–12 years (33), with high levels of internal consistency (*α* = 0.80) and test–retest reliability (*r* = 0.92). The SEQ yields 4 mean scale scores: (1) hyper-responsiveness, (2) hypo-responsiveness, (3) sensory interests, repetitions, and seeking behaviors, and (4) enhanced perception. For the purposes of this study, only the SEQ hyper-responsiveness scale was used as a measure of sensory over-responsivity.

Irritability was measured with the Aberrant Behavior Checklist [ABC (34)]. The ABC is a caregiver report measure commonly used in clinical trials for autism interventions to assess the severity of specific behaviors in several domains, including irritability/agitation/crying, social withdrawal, stereotypic behavior, hyperactivity/non-compliance, and inappropriate speech.

#### 2.2.2 Co-occurring anxiety and ADHD

Anxiety and ADHD diagnoses and symptom severity were assessed with the Preschool Age Psychiatric Assessment [PAPA (35)], a caregiver interview that assesses for psychopathology in young utilizing criteria based on the Diagnostic and Statistical Manual Version IV [DSM IV (36)]. Previous studies have used the PAPA to assess co-occurring psychiatric disorders in autistic children (65). The PAPA includes assessment of most DSM IV diagnostic criteria insofar as they are relevant to younger children, plus all items in the Diagnostic Classification: 0–3R (35) and is adapted from the parent-version of the Child and Adolescent Psychiatric Assessment [CAPA (37)], which has also previously been used to assess co-occurring psychiatric disorders in autistic populations (38). The PAPA employs a structured protocol with specific questions and probes that ensure interviewees (1) understand the question being asked; (2) provide clear information on behavior or feelings relevant to the symptom; and (3) report the symptom at a pre-specified level of severity as defined in an extensive glossary. When symptoms are reported, their frequency, duration and dates of onset are also collected, to determine whether they meet the symptom overlap and duration criteria for the various DSM IV diagnoses. A three month “primary period” is used rather than a longer period, because shorter recall periods are associated with more accurate recall (37, 38). Test-retest studies show reliability comparable to interviews for older populations, with kappas ranging from 0.36 to 0.79 and intraclass correlations for DSM IV scales from 0.56 to 0.90 (39).

In the current study, anxiety disorders and ADHD were assessed both at the symptom severity level and diagnostic level. Children were included in the anxiety disorder group if they met diagnostic criteria for generalized anxiety disorder (GAD), separation anxiety disorder (SAD), and/or social phobia disorder. Similarly, the designation of ADHD was based off meeting symptom, duration, and impairment criteria for ADHD. ADHD diagnoses were not subdivided by ADHD type (inattentive, hyperactive, combined). Overall, 49 children (71%) met criteria for a co-occurring anxiety disorder (with or without co-occurring ADHD) and 42 children (61%) met criteria for co-occurring ADHD (with or without a co-occurring anxiety disorder). Most children had both ADHD and an anxiety disorder (45%) rather than a single co-occurring condition (39%). Of those with a single condition, 26% met criteria for an anxiety disorder only and 11% met criteria for ADHD only. Only 9 children (13%) did not have a co-occurring disorder. Diagnostic breakdowns are summarized in Table 1.

The prevalence of anxiety in this cohort was notably higher than typically reported in this age group which may be due to our inclusion of anxiety disorders beyond generalized anxiety disorder (GAD). However, these findings are similar to studies of that include phobias in addition to GAD (40, 41).

#### 2.2.3 Executive function

General intellectual functioning was assessed for most participants using the Abbreviated Battery Intelligence Quotient (ABIQ) from the Stanford-Binet Intelligence Scales, Fifth Edition [SB5 (42)]. The ABIQ has been shown to closely approximate full-scale IQ in autistic children with adequate expressive/receptive language skills and attentional/behavioral regulation (43). In children who were anticipated to not have the language skill or behavioral regulation to fully participate in the ABIQ, general intelligence functioning was measured via the Mullen Scale of Early Learning, American Guidance Service Edition [MSEL:AGS (44)] which includes nonverbal and play-based tasks. Developmental quotient (DQ) was calculated by dividing developmental age by chronological age.

Executive function domains were measured via the Behavior Rating Inventory of Executive Function, Preschool Version [BRIEF-P (45, 46)]. BRIEF-P is a 63-item caregiver report measure of executive functioning for young children and is validated for ages 2 years to 5 years 11 months. Frequencies of behaviors are rated on a three-point Likert scale (1 = never, 2 = sometimes, 3 = often) which correlates to specific indexes (inhibitory self-control, flexibility, and emergent metacognition) and subscales (inhibit, shift, emotional control, working memory, and plan/organize) that are compared to normative data. Standard scores were utilized in analysis.

### 2.3 Analyses

General linear models were used to examine the unique effects of a co-occurring anxiety disorder and/or ADHD on core and associated autistic features, as well as executive functioning difficulties. Anxiety disorder and ADHD designations were input separately into the model as dichotomous variables (present vs. absent). This method provides marginal predictions for both an anxiety disorder and ADHD while accounting for non-independence between groups, given that some children have both an anxiety disorder and ADHD. As such, this identifies the unique contribution of anxiety disorders and ADHD diagnoses on each outcome.

Mediation models tested the hypothesis that symptom levels of anxiety and ADHD influence the core and associated features of autism through differences in executive function. The mediation models exploring the relationship between anxiety symptoms, executive function, and autism features included ADHD symptoms as a covariate. Similarly, the mediation models exploring the relationship between ADHD symptoms, executive function, and autism features included anxiety symptoms as a covariate.

There was no significant difference in age or developmental quotient (DQ) when comparing children with and without ADHD or children with and without anxiety disorders. Thus, age and DQ were not utilized in the models or analyses (47). Comparison statistics are summarized in Table 2.

**Table 2:** Comparison of age and DQ by ADHD and anxiety status.

Comparison Group	Variable	t(df) = 67	*p*-value
ADHD vs. no ADHD	DQ	0.07	0.94
ADHD vs. no ADHD	Age	−1.9	0.07
Anxiety disorder vs. no anxiety disorder	DQ	−.077	0.44
Anxiety disorder vs. no anxiety disorder	Age	−1.41	0.16

DQ, developmental quotient; ADHD, attention-deficit hyperactivity disorder; t(df), t-value(degrees of freedom).

All models were run in SAS. Mediation analyses were assessed using the PROCESS macro version 3.0 for SAS (http://www.processmacro.org), with statistical significance of the indirect effect assessed using a bootstrapping procedure with 10,000 bootstrap samples and 95% confidence intervals estimation.

## 3 Results

### 3.1 Co-occurring disorders and autistic features

After accounting for ADHD, a co-occurring anxiety disorder was linked to an overall increase in autistic features [*F*(1) = 4.6, *p* < .05] as measured by the ADOS-2. A co-occurring anxiety disorder was also associated with increased ritualistic [*F*(1) = 10.2, *p* < .01] and sameness [*F*(1) = 3.9, *p* = .05] behaviors, as well as heightened sensory hyper-responsivity [*F*(1) = 16.5, *p* < .001]. Conversely, when accounting for a co-occurring anxiety disorder, co-occurring ADHD was associated with increased levels of self-injurious behaviors [*F*(1) = 4.4, *p* < .05] and increased levels of irritability [*F*(1) = 0.89, *p* = 0.4], but it was not associated with an overall increase of autistic core features. Neither ADHD nor anxiety disorders were associated with the ADOS-2 social affect score or the RBS-R stereotyped motor behaviors, compulsive behaviors, or restricted behaviors. Results of core and associated behavioral features associated with an anxiety disorder and/or ADHD are summarized in Table 3.

**Table 3:** Autism features associated with co-occurring conditions.

Behavioral domain	Measurement	Anxiety	ADHD
Overall autism features	ADOS-2	*F*(1) = 4.6, *p* < 0.05	N.S.
Social affect score	ADOS-2	N.S.	N.S.
Sensory hyper-responsivity	SEQ	*F*(1) = 16.5, *p* < 0.001	N.S.
Restricted and Repetitive Behaviors			
Ritualistic	RBS-R	*F*(1) = 10.2, *p* < 0.01	N.S.
Sameness	RBS-R	*F*(1) = 3.9, *p* < 0.05	N.S.
Self-injury	RBS-R	N.S.	*F*(1) = 4.4, *p* < 0.05
Stereotyped motor behavior	RBS-R	N.S.	N.S.
Compulsive behaviors	RBS-R	N.S.	N.S.
Restricted behaviors	RBS-R	N.S.	N.S.
Irritability	ABC	N.S.	*F*(1) = 7.3, *p* < 0.01

ASD, Autism spectrum disorder; ADHD, attention-deficit hyperactivity disorder; ADOS-2, Autism diagnostic observation schedule – second edition; RBS-R, restricted and repetitive behaviors scale; SEQ, sensory experiences questionnaire; ABC, Aberrant behavior checklist; N.S., not significant.

### 3.2 Co-occurring disorders and executive function difficulties

In terms of executive function (Table 4), when accounting for ADHD, children with a co-occurring anxiety disorder had greater reported difficulty with attention shifting [*F*(1) = 22.6, *p* < .0001] and emotional control [*F*(1) = 3.4, *p* = 0.07] on the BRIEF-P. When accounting for an anxiety disorder, co-occurring ADHD was linked to greater difficulty with inhibition [*F*(1) = 12.2, *p* < .001], working memory[*F*(1) = 8.7, *p* < .01], and planning/organizing [*F*(1) = 8.1, *p* < .01]. Additionally, there was an interaction effect between anxiety disorders and ADHD on inhibition [*F*(1) = 4.68, *p* < .05], attention shifting [*F*(1) = 4.91, *p* < .05], and emotional control [*F*(1) = 7.61, *p* < .01].

**Table 4:** Executive function domains correlated with co-occurring conditions.

Executive function domain^a^	Anxiety	ADHD	Anxiety*ADHD interaction
Inhibition	N.S.	*F*(1) = 12.2, *p* < 0.001	*F*(1) = 4.7, *p* = 0.03
Shifting Attention	*F*(1) = 22.6, *p* < 0.0001	N.S.	*F*(1) = 4.9, *p* < 0.05
Emotional Control	*F*(1) = 3.4, *p* = 0.07	*F*(1) = 7.5, *p* < 0.01	*F*(1) = 7.6, *p* < 0.01
Working Memory	N.S.	*F*(1) = 8.6, *p* < 0.005	N.S.
Planning/Organizing	N.S.	*F*(1) = 8.1, *p* < 0.01	N.S.

BRIEF-P, behavior rating inventory of executive function, preschool version; N.S., not significant.

^a^ From BRIEF-P subscores.

### 3.3 Executive function as a mediator of differences in how anxiety and ADHD symptoms impact core and associated autistic features

**Figure 1:**
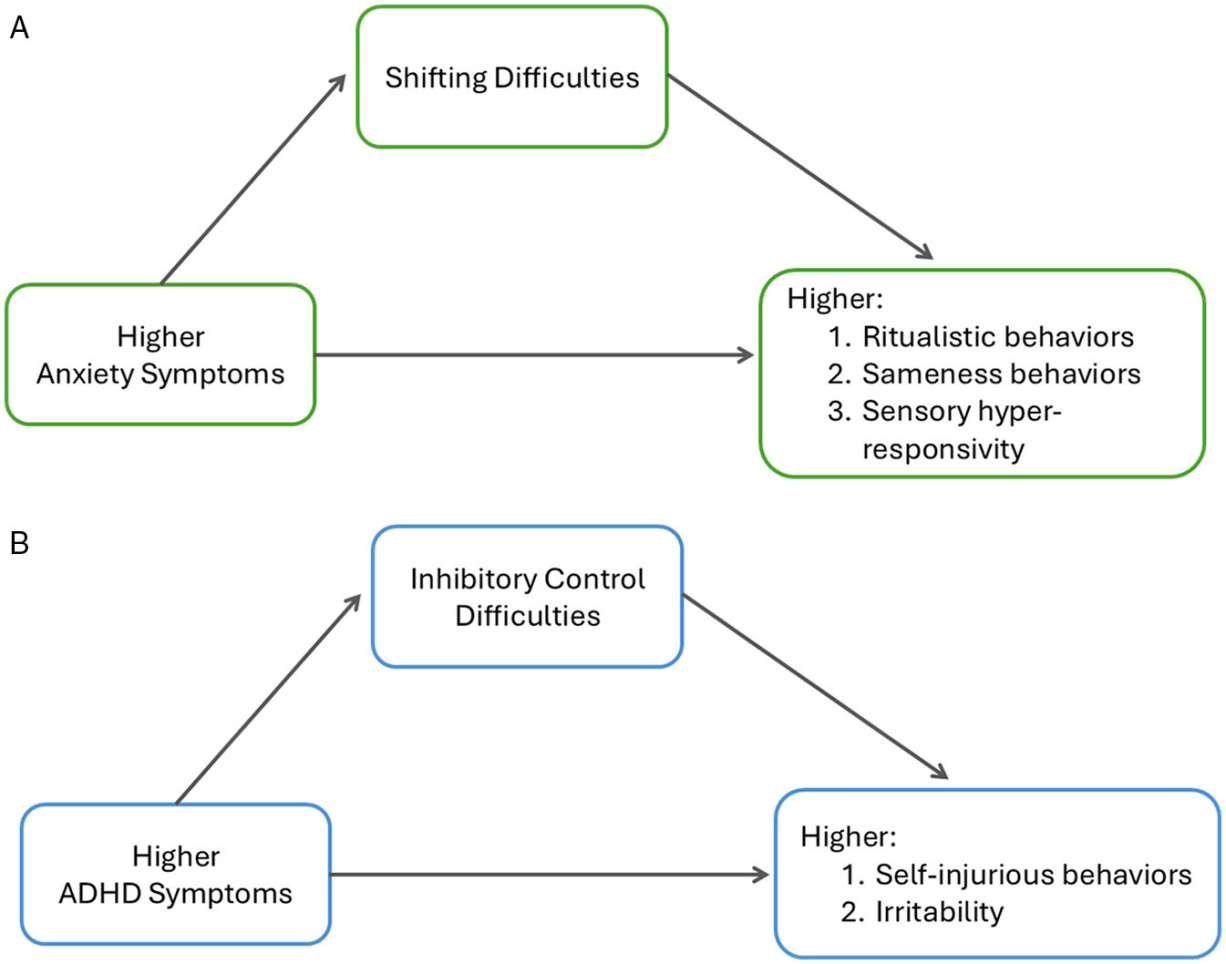
**(A)** Mediation path diagram between anxiety symptoms, executive function, and autism symptoms. Data are presented as ß(SE). In children with anxiety, when accounting for co-occurring ADHD, attention shifting difficulties mediate the relationship between higher anxiety symptoms and more difficulties with ritualistic behaviors [*β*(SE), = 0.22(0.1), 95% CIs = 0.05–0.5], more sameness behaviors [*β*(SE) = 0.35(0.12), 95% CIs = 0.11–0.59], and higher levels of sensory hyper-responsivity (*β*(SE). **(B)** Mediation path diagram between ADHD symptoms, executive function, and autism symptoms. Data are presented as ß(SE). In children with ADHD, when accounting for co-occurring anxiety, inhibition difficulties mediate the relationship between higher ADHD symptoms and more self-injurious behaviors [*β*(SE) = 0.37(0.17), 95% CIs = 0.08–0.74] and irritability [*β*(SE) = 0.10(0.05), 95% CIs = 0.02–0.2].

Figure 1 summarizes the findings of the mediation analyses. When accounting for co-occurring ADHD symptoms, children with autism and higher anxiety symptoms who have the highest levels of shifting difficulties (Figure 1A), have more ritualistic behaviors [β(SE) = 0.22(0.1), 95% CIs = 0.05–0.5], more sameness behaviors [β(SE) = 0.35(0.12), 95% CIs = 0.11–0.59], and higher levels of sensory hyper-responsivity [β(SE) = 0.99(0.45), 95% CIs = 0.11–1.9]. On the other hand, when accounting for anxiety symptoms, children with autism and ADHD symptoms who have greater difficulties with inhibitory control (Figure 1B) have higher levels of self-injurious behaviors [β(SE) = 0.37(0.17), 95% CIs = 0.08–0.74] and irritability [β(SE) = 0.10(0.05), 95% CIs = 0.02–0.2].

## 4 Discussion

This study supports the hypothesis that co-occurring ADHD and anxiety differentially affect the core and associated behavioral features of autism and executive function abilities in preschoolers. It is important to note that while this study design does not elucidate the directionality or causality between these conditions, it does support growing evidence that these conditions can influence each other. These findings offer insights into the complex interplay of these conditions in early childhood.

By using an additive main effect general linear model, we identified unique associations between the presence of an anxiety disorder and/or ADHD on specific core and associated features of autism. This analysis also revealed unique executive function differences, with difficulties in attention shifting being associated with an anxiety disorder while difficulties with inhibition were associated with ADHD. A mediation analysis at the symptom level examined the influence of these specific executive function differences in mediating autism features.

Our findings showed that having an anxiety disorder, but not ADHD, is associated with increased overall autism-related behavioral features. Specifically, having an anxiety disorder was associated with increased ritualistic behaviors and need for sameness It is important to note that there may be multiple complex transdiagnostic interactions between these behaviors that should be further elucidated (48). Interestingly, an experimental study found that placing people in anxiogenic situations increased their ritualistic and repetitive behaviors. These behaviors may play a role in alleviating or reducing an internal anxiety state. It is also possible that increased rigidity and cognitive inflexibility may lead to a greater likelihood of developing an anxiety disorder. Intolerance of uncertainty is associated with higher levels of anxiety in autistic individuals (49).

Alternatively, increased ADHD symptoms were associated with increased self-injurious behavior, suggesting a distinct pattern of correlation between the presence of ADHD and autistic features. The presence of co-occurring ADHD was also associated with higher levels of irritability, whereas a co-occurring anxiety disorder was associated with greater sensory hyper-responsivity. These findings were interesting as prior research has found atypical sensory profiles in people with ADHD or impaired attention regardless of whether co-occurring autism is present (50, 51).

In terms of executive function, children with co-occurring anxiety disorders had more difficulty with attention shifting compared to children with ADHD, while children with ADHD had more difficulty with inhibition compared to children with anxiety disorders. Autistic children with both an anxiety disorder and ADHD had difficulties across both inhibition and attention shifting, as well as emotional control. These findings have several important clinical implications. They highlight the need for a nuanced approach to assessment and intervention in young autistic children who present with co-occurring ADHD and/or anxiety disorders. There is evidence that co-occurring conditions can affect intervention outcomes. Different pharmacologic management of ADHD in autistic individuals has been recommended due to decreased tolerability and efficacy of some medications (52). Given that these co-occurring conditions can uniquely impact the presentation of autism features, tailored interventions that address both autism-specific behaviors and the symptoms of ADHD and anxiety disorders may be more effective.

The effectiveness of various psychotherapies for treating co-occurring conditions in autistic children has shown variable results and appears to be affected by the specific conditions and interventional targets. Notably, cognitive behavioral therapy (CBT) tailored for anxiety has been effective in reducing anxiety symptoms in autistic children. Protocols such as the Behavioral Interventions for Anxiety in Children with Autism (BIACA) have demonstrated superior outcomes compared to traditional CBT (53–55) in terms of reduction of anxiety symptoms. BIACA modifies traditional CBT for anxiety by including divided sessions between the child and their parents, integrating social communication skills, and employing special interests as part of a reward system.

Research on BIACA and similar modified CBT approaches suggest that co-occurring ADHD does not generally impair a treatment's effectiveness for anxiety (56). However, some adjustments, such as shortening sessions, have been suggested to better accommodate children with ADHD (57). Interestingly, some studies report that autistic children with co-occurring ADHD may derive more benefit from these anxiety treatments than those without ADHD (58). Mediation analyses conducted during treatment of anxiety disorders have shown that as anxiety symptoms improve, there is a reduction in restricted and repetitive behaviors as well as improvements in social communication (59).

In contrast, group interventions focused on social skills in autistic children have shown improvements in children without a co-occurring condition and with co-occurring anxiety disorders but have been less effective for those with ADHD (60). It is possible the group format differentially affects children with ADHD.

Another clinical implication of the present findings is the importance of considering executive functioning in the evaluation and intervention of young autistic children. Since executive function difficulties, particularly related to attention shifting and inhibition, mediate the relationship between co-occurring conditions and autism features, interventions aimed at improving executive function may help mitigate some of the negative impacts of anxiety and ADHD on core autism features. Notably, children with anxiety who were responders to CBT were found to have improved attention shifting while non-responders did not (61). Additionally, mindfulness-based therapies reduce inattention in ADHD (62).

These findings underscore the critical need for identifying clinically significant subgroups within the autistic population and targeting interventions appropriately, especially from an early age.

### 4.1 Limitations

The current study was cross-sectional in design and thus we are unable to determine the temporality of the relationships between executive difficulties and the impact of co-occurring conditions on core and associated autistic features. Future prospective studies should explore the developmental time course of these associations.

Diagnosis of co-occurring conditions in autistic individuals using measures designed for non-autistic children can be difficult due to overlap in similar behavioral features, atypical presentation, communication barriers, and behavioral context. The PAPA used in this study was designed for use in non-autistic children, which may not accurately reflect the nuances in co-occurring conditions in autistic individuals. Future studies would benefit from using measures validated in this population such as the Anxiety and Related Disorders Interview Schedule, Autism Spectrum Addendum [ADIS/ASA (63)].

It is important to note that the caregiver report measures of this study may not represent the experiences of the children or their abilities but rather how their caregiver perceives the children's abilities in these areas. Additionally, the BRIEF-P, which was used to measure executive functioning in the current study, assesses the impact of executive difficulties on everyday functioning, which may not be directly related to core executive functioning skills. As such, further studies would benefit from objective task-based measurements of executive function.

## 5 Conclusion

Overall, our findings support the growing literature body that highlights the importance of considering co-occurring conditions in autistic individuals. This study provides evidence that co-occurring anxiety and ADHD distinctly influence the presentation of autism in young children with executive function playing a mediating role. This emphasizes the diverse experiences and behavioral profiles of autistic individuals. By studying young children it also provides potential areas for early intervention. Given the early emergence of executive function differences, interventions targeting executive functions, such as improving cognitive flexibility and inhibition, may have benefits beyond those areas alone. Future research should explore the longitudinal relationship between these commonly co-occurring conditions and how interventions in executive function might influence outcomes over time.

## Data Availability

The raw data supporting the conclusions of this article will be made available by the authors, without undue reservation.
